# Mesenchymal Stem Cells Derived from Adipose Tissue vs Bone Marrow: *In Vitro* Comparison of Their Tropism towards Gliomas

**DOI:** 10.1371/journal.pone.0058198

**Published:** 2013-03-12

**Authors:** Courtney Pendleton, Qian Li, David A. Chesler, Kristy Yuan, Hugo Guerrero-Cazares, Alfredo Quinones-Hinojosa

**Affiliations:** The Johns Hopkins Medical Institutes, Departments of Neurosurgery and Oncology, Baltimore, Maryland, United States of America; University of Pécs Medical School, Hungary

## Abstract

**Introduction:**

Glioblastoma is the most common primary malignant brain tumor, and is refractory to surgical resection, radiation, and chemotherapy. Human mesenchymal stem cells (hMSC) may be harvested from bone marrow (BMSC) and adipose (AMSC) tissue. These cells are a promising avenue of investigation for the delivery of adjuvant therapies. Despite extensive research into putative mechanisms for the tumor tropism of MSCs, there remains no direct comparison of the efficacy and specificity of AMSC and BMSC tropism towards glioma.

**Methods:**

Under an IRB-approved protocol, intraoperative human Adipose MSCs (hAMSCs) were established and characterized for cell surface markers of mesenchymal stem cell origin in conjunction with the potential for tri-lineage differentiation (adipogenic, chondrogenic, and osteogenic). Validated experimental hAMSCs were compared to commercially derived hBMSCs (Lonza) and hAMSCs (Invitrogen) for growth responsiveness and glioma tropism in response to glioma conditioned media obtained from primary glioma neurosphere cultures.

**Results:**

Commercial and primary culture AMSCs and commercial BMSCs demonstrated no statistically significant difference in their migration towards glioma conditioned media *in vitro*. There was statistically significant difference in the proliferation rate of both commercial AMSCs and BMSCs as compared to primary culture AMSCs, suggesting primary cultures have a slower growth rate than commercially available cell lines.

**Conclusions:**

Adipose- and bone marrow-derived mesenchymal stem cells have similar *in vitro* glioma tropism. Given the well-documented ability to harvest larger numbers of AMSCs under local anesthesia, adipose tissue may provide a more efficient source of MSCs for research and clinical applications, while minimizing patient morbidity during cell harvesting.

## Introduction

### Background

Glioblastoma (GB) is the most common primary malignant brain tumor, and is refractory to surgical resection, radiation, and chemotherapy [1]. This is likely due to the presence of micrometastatic nests of glioma cells at sites distant from the main tumor mass as well as stem cell-like subpopulations, referred to as brain tumor stem cells (BTSCs) which are known to be refractory to clinically relevant doses of chemo- and radiation therapy [2]–[5]. The poor prognosis has served as the impetus for developing new therapeutic modalities targeting the micrometastatic nature of this disease.

### Mesenchymal Stem Cell Therapeutic Usage

Human stem cells have shown promise as a therapeutic approach to brain cancer. Human neural stem cells (hNSC) selectively migrate to malignant gliomas *in vivo* [6] and *in vitro* [6], and have been used to deliver cytotoxic [7] and immunomodulatory [8] therapies. The translational potential of hNSCs is limited by practical difficulties in harvesting and expanding hNSCs *ex vivo* [9].

Human mesenchymal stem cells (hMSC) exhibit selective tropism similar to hNSCs [10], [11], migrating significant distances to target gliomas [12]–[14]. hMSCs can be readily harvested from a broad range of sources, including adipose tissue [15], [16], bone marrow [17], umbilical cord [18], [19], and dental pulp [20]–[22]. Adipose- (AMSC) and bone marrow-derived MSCs (BMSC) have been most extensively evaluated as they offer the most accessible source of MSC for use in research and future clinical applications. hMSCs have been used as a delivery vehicle for therapeutic molecules in human gliomas [9], [23], [24], colon cancer [25], breast cancer [26], ovarian cancer [27], melanoma [28], and prostate cancer [29]; in these studies, hMSCs homed to the tumor site *in vitro*, and demonstrated an anti-tumor effect *in vivo*.

Adipose tissue is less invasive and less expensive than bone marrow to obtain [30], [31]. Additionally, unmodified hAMSCs remain free of oncogenic transformation for at least eight months, when injected into immunocompromised mice, demonstrating more oncogenic resistance than BM-MSCs [32]. The possibility of malignant transformation of transplanted MSCs remains a serious, albeit controversial, topic among researchers [33]. Recent literature indicates that reports of malignant or oncogenic transformation of MSCs may reflect the role of cell culture cross-contamination rather than true transformation [34].

### Previous Direct Comparisons in Clinical Applications

MSCs from the two sources (derived from bone marrow and adipose) have been compared in models of ischemia [35]. Notably, cell proliferation rates were used to conclude that AMSC would be more efficacious in clinical application than BMSC, as MSC from adipose tissue had a significantly shorter doubling time [35]. The use of MSCs for the treatment of gliomas requires a population of cells with a doubling time and proliferation rate rapid enough to allow timely expansion of autologous cells for clinical applications. Harvest efficiency for AMSCs and BMSCs partially depends on donor age [36]; the BMSC population has been shown to decrease substantially with age, casting further doubt on the use of autologous BMSCs as a therapeutic delivery vehicle in the patient population most often afflicted with gliomas [37].

No literature exists comparing the efficacy and specificity of AMSC and BMSC glioma tropism. This study presents the first direct comparison of *in vitro* glioma tropism for MSCs from two distinct tissue sources as well as presenting data for the establishment of hAMSCs from intraoperative tissue and the tropism of these early passaged stem cells towards gliomas. The results demonstrate that both AMSC and BMSC have similar glioma tropism, while AMSC may be harvested more easily in greater numbers from patients; this new information may help direct the course of future research into MSCs as a therapeutic delivery vehicle for the treatment of glioblastoma.

## Materials and Methods

### Ethics statement

Intraoperative surgical samples were obtained under surgical written consent according to National Institutes of Health Institutional Review Board Exempt. Under Johns Hopkins University approved protocols, based on its designation as pathological waste. All animal procedures were reviewed and approved by the Ethical Committee for Use of Laboratory Animals of the Johns Hopkins University guidelines.

### Cell Culture and Isolation of primary hAMSCs and BTSCs

Commercially available hAMSCs were purchased from Invitrogen. Invitrogen harvested each lot of AMSC from a single donor using lipoaspirate, and the cells were expanded for one passage prior to cryopreservation and shipment. Primary hAMSC cultures were established under an approved IRB protocol using excess tissue that would otherwise have been discarded from adipose explants obtained from patients expected to require a graft for repair of cerebrospinal fluid leak during intracranial operations after informed consent was provided. Once obtained, adipose tissue was stored on ice; under sterile cell culture conditions the adipose tissue was separated using manual dissociation and enzymatic digestion with collagenase. The dissociated tissue was centrifuged for 5 minutes, 500 G; the supernatant was decanted, and the vascular-stromal fraction resuspended and plated in six well plates at a density of 5 grams of starting tissue per well

Both commercial and primary AMSC lines were cultured in MesenproRS media (Invitrogen) supplemented with 1× GlutaMax (Invitrogen) and 1% antibiotic/antimycotic (Invitrogen) and maintained in an incubator at 37°C/5% CO_2_. The media was changed every three days, and the cells were split at 80–90% confluence. The cells were used at early passage (<5 passages) for all experiments.

Commercially available BMSCs were purchased from Lonza and maintained as adherent cultures in complete Mesenchymal Stem Cell Growth Medium (MSCGM BulletKit; Lonza) at 37°C/5% CO_2_. Media was changed every two days, and the cells were split when they reached 80–90% confluence. The cells were used at early passage (<5 passages) for all experiments.

Following previously published techniques [38], glioma stem cell lines were established under an IRB approved research protocol using surgical explants from patients undergoing operative resection of intracranial glioma after informed consent was provided. Briefly, under sterile conditions, surgical glioma explants were separated by manual dissociation and centrifuged after which cell pellets were resuspended in growth medium comprised of DMEM/F12 (Invitrogen) supplemented with B27 growth supplement (Gibco), antibiotic/antimycotic (Gibco), FGF (20 µg/ml; Peprotech) and EGF (20 µg/ml; Peprotech) and subsequently plated onto laminin-coated tissue culture flasks at a initial density of 2500 cells/cm^2^. The media was changed every three days, and cells were split at 80–90% confluence. The cells were used at early passage (<12 passages) for all experiments. For experiments requiring media without additional growth factors, all cell lines were treated with DMEM: F12 with antibiotics without additional growth factors.

### Generation of Glioma Conditioned Media

Glioma conditioned media was produced in the following manner. Glioma cells were plated in a six well plate previously coated with laminin solution as described above, at a density of 250,000 cells per well. Cells were allowed to adhere and proliferate for twenty-four hours; after that time, the complete media was removed, the cells were twice washed with PBS, and 3 mL of serum free media was added to each well. After 48 hours, the media was harvested and centrifuged; the supernatant was then used as glioma conditioned media in these experiments.

### Differentiation protocol

Primary AMSC lines were differentiated using commercially available differentiation media kits (StemPro Adipogenesis, Chondrogenesis and Osteogenesis Kits; Invitrogen). Cells were seeded at densities of 2.1×10^4^ (adipocyte), 4.2×10^3^ (osteocyte) cells/cm^2^; for chondrocyte differetiation, cells were seeded at 2.5×10^5^ cells in a 15 mL conical tube (Falcon). The differentiation was conducted for a three-week period, in accordance with the manufacturer instructions. Media was changed every 3 days. For adipocyte differentiation, Oil Red O stain was prepared with isopropanol and water, then filtered; differentiated cells were fixed in 4% PFA, rinsed with isopropanol, and stained with Oil Red O for 15 minutes; cells were rinsed with isopropanol, then counterstained with hematoxylin for 5 minutes. For osteocyte differentiation, 2% Alizarin Red S stain was prepared with water, then filtered; differentiated cells were fixed in 4% PFA, rinsed with PBS, then stained with Alizarin Red S for 5 minutes. For chondrocyte differentiation, immunohistochemistry for Collagen II staining was performed using paraffin embedded differentiated cells, a Rabbit polyclonal Collagen II primary antibody (AbCam), and a biotinylated goat anti-rabbit secondary antibody (Vector Lab). Following staining, cells were imaged with light microscopy. Differentiation protocols were adapted from the commercial differentiation media kits (StemPro; Invitrogen), and the published literature, in particular Bunnell et. al. [39].

### Cytometry

For commercial and primary AMSC lines, 1×10^5^ cells were incubated in 7 mL blocking solution (10%FBS in 1% BSA/PBS) for 10 minutes, centrifuged at 400 G/4°C/5 minutes, and resupended in 100 uL buffer (1% BSA/PBS) for each condition. Primary conjugated antibodies were added at an amount of 5 µL/1×10^6^ cells for CD73, and 20 µL/1×10^6^ cells for CD31, CD45, CD105, and CD90. Cells were incubated for 30 minutes, washed with PBS, and centrifuged at 400 G/4°C/5 minutes; this was repeated for a total of three washes. Cells were resuspended in 700 µL PBS and transferred to polypropylene tubes with mesh tops before using a 4 channel flow cytometer (FacsCalibur) to acquire 20,000 events for analysis.

### Assessment of Invasive Capacity

BTSCs invasion capacity was evaluated through transwell migration using modified Boyden chamber plates with matrigel-coated inserts (BD BioCoat Matrigel Invasion Chambers in two 24-well plates, 8.0 µm; BD Biosciences). The matrigel was rehydrated with 500 µL complete media and incubated at 37°C/5% CO_2_ for 4 hours. The media was aspirated, and cells seeded in their respective complete media at a density of 15,000 cells per insert and allowed to adhere overnight at 37°C/5% CO_2_. Following this the complete media was aspirated, and 500 uL of serum free media (DMEM: F12 with antibiotics) was placed in the chambers; the cells were allowed to serum starve for 4 hours. After serum starvation, the following experimental conditions were established in the bottom wells (n = 9): serum free media, 10% FBS, and conditioned media produced using two separate glioma cell lines, as described above Plates were incubated in these conditions at 37°C/5% CO_2_ for 24 hours after which the upper chambers were removed, and the inserts stained with the DiffQuick staining kit and mounted onto glass slides.

Results of invasion assays were determined by a third-party blinded to the experimental and control conditions counting cells within nine fields using a protocol with set coordinates used for all invasion assays in our laboratory. For comparison within individual cell lines, the migrating cells were normalized to the serum free media condition.

### Growth curve assays

Cell number was evaluated on days 0, 2, 4, 6, 8, and 10 using trypan blue exclusion method on an automated cell counter (ViaCell).

### Statistical analysis

Statistical analysis was carried out using GraphPad Prism software. The results from the modified Boyden chamber assays were analyzed using Student's t-tests (two-tailed), and the growth curve assays were analyzed using a repeated measures one-way ANOVA with Tukey's multiple comparison post-hoc test.

## Results

### Characterization of Stem-like Characteristics in Commercial and Experimental MSCs

**Figure 1:**
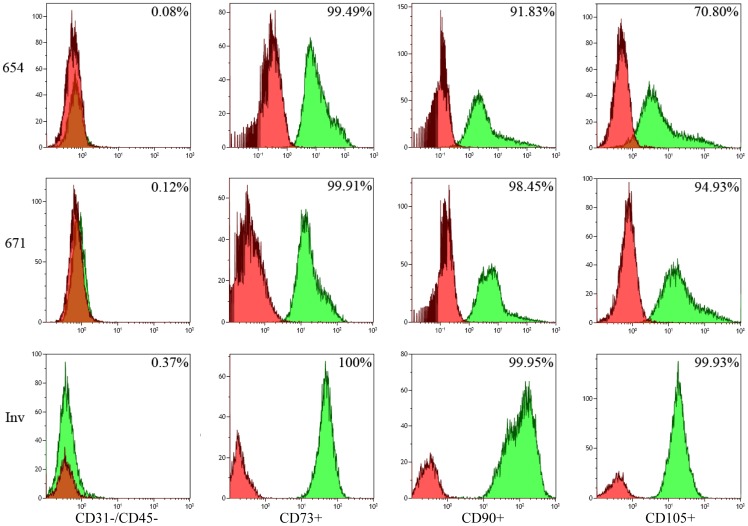
Stemness of the primary adipose-derived mesenchymal stem cell lines (AMSC), and the Invitrogen supplied commercial AMSC line, confirmed with FACS documenting the absence of hematopoetic surface antigens CD31/CD45 and the presence of cell adhesion markers CD73/CD90/CD105. Percentage of each antigen was analyzed using Kaluza software and labeled in the responsive graph.

**Figure 2:**
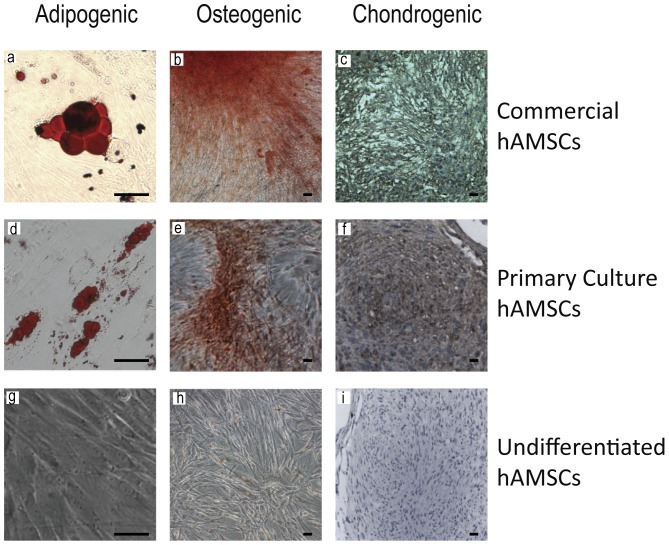
Stemness of primary AMSC lines demonstrated with differentiation along three mesenchymal lineages, Adipocyte (a, d [48], g), Osteocyte (b [48], e, h), and Chondrocyte (c [48], f, i), documented via lineage specific staining with Oil Red O, Alizarin Red, and Collagen II, respectively. The details of the staining protocols are provided in the methods section. Scale bar, 50 µm.

The presence of mesenchymal lineage-associated cell surface markers of AMSC primary cell lines was confirmed with FACS analysis for the presence of cell adhesion markers CD73 (AMSC primary line 654, AMSC primary line 671 and commercial AMSC line; 99.49%, 99.91% and 100%)/CD90 (AMSC primary line 654, AMSC primary line 671 and commercial AMSC line; 91.83%, 98.45% and 99.95%)/CD105 (AMSC primary line 654, AMSC primary line 671 and commercial AMSC line; 70.80%, 94.93% and 99.93%) and the absence of hematopoetic surface antigens CD31/CD45 (AMSC primary line 654, AMSC primary line 671 and commercial AMSC line; 0.08%, 0.12% and 0.37%) (**Figure 1**). Pleuripotentiality for these lines was confirmed through differentiation along three mesenchymal lineages: adipocytes, osteocytes, and chondrocytes (**Figure 2**).

The stemness of commercially obtained BMSC and AMSC lines was confirmed by the respective commercial providers through FACS and differentiation assays. There was no appreciable difference in cell morphology between primary and commercial AMSC lines and the commercial BMSC line (not shown).

### Invasive Capacity

**Figure 3:**
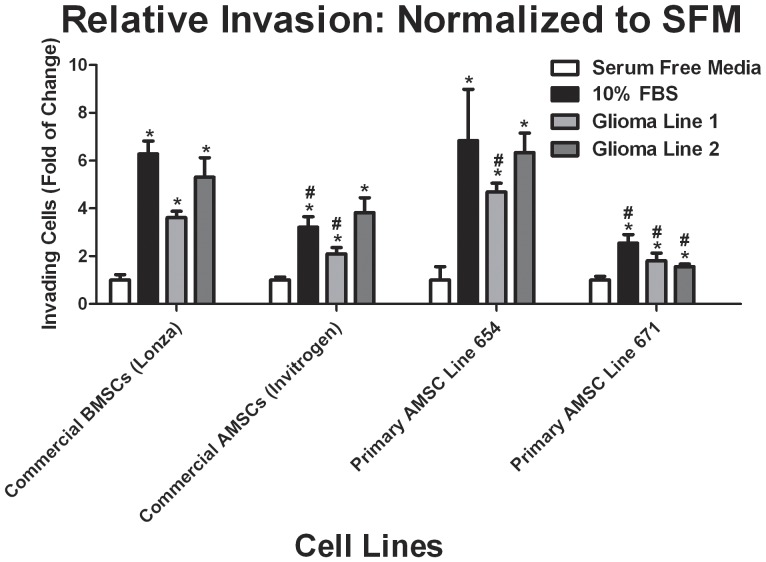
Primary and commercial AMSCs and commercial BMSCs have similar tropism to glioma conditioned media *in vitro*, although there is notable variability in the tropism of the primary AMSC lines. Analysis of individual cell line tumor tropism in a matrigel-coated insert Boyden chamber migration assay; migration for each line was normalized to the serum free media condition. Results are reported as mean ± S.E.M., n = 9. (* represents the statistically significant difference within groups and # represents the statistically significant difference between groups).

The relative capacity of migration and invasion in response to glioma-secreted chemotactic factors for BMSCs compared with AMSCs was assessed using matrigel transwell invasion assays. In all four cell lines, both BMSCs and AMSCs migrated towards glioma conditioned media in numbers greater than the background cell motility (**Figure 3**). Variation in the robustness of this migration was seen between cell lines, with statistical significance noted in BMSC migration toward glioma conditioned media (glioma lines 1 and 2 CM; p<0.001 for both conditions), as well as the AMSC primary line 654 (glioma 1 and 2 CM; p<0.01 and p<0.001 respectively), AMSC primary line 671 (glioma 1 and 2 CM; p<0.05 for both conditions), and the commercial AMSC line (glioma lines 1 and 2 CM; p<0.01 and p<0.001 respectively)(**Figure 3**). Additionally, three AMSC lines were compared to BMSCs for each group and found significant differences from commercial BMSC line with commercial AMSC line (10% FBS and glioma line 1 CM; p<0.01), AMSC primary line 654 (glioma line 1 CM; p<0.05), and AMSC primary line 671 (10% FBS and glioma lines 1 and 2 CM; p<0.01) ( **Figure 3**). Although AMSC lines were found have different tropism capacities with BMSC line, both AMSC primary lines -, commercial AMSCs, and commercial BMSCs all demonstrated statistically significant preferential migration to both glioma 1 and 2 conditioned media indicating that AMSCs (primary and commercial) have similar tropism to glioma-secreted factors as well as BMSCs.

### Proliferative Capacity

**Figure 4:**
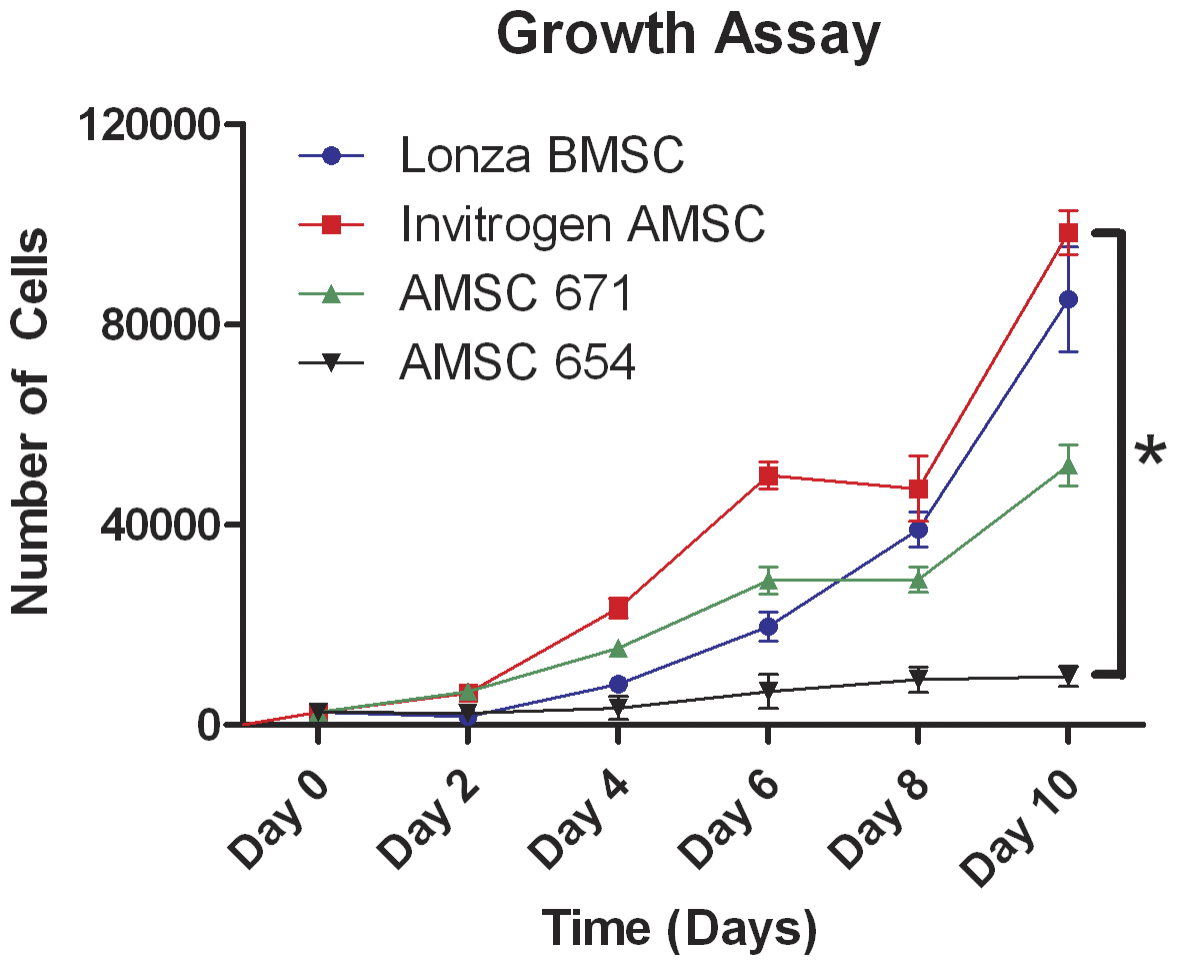
Growth curve documenting relative proliferation rates of commercial (Lonza) BMSC, commercial (Invitrogen) AMSC, and the two primary AMSC cell lines (671, 654). The values shown are the mean of three replicates per cell line per time point, and the S.E.M (*:p<0.05).

To examine the relative growth capacities of BMSCs versus AMSCs, growth curves of were performed with cells grown at 37°C/5% CO_2_ in their native media over a 10 day period. A statistically significant difference in proliferation rate was found between commercial AMSC line and AMSC primary line 654 over 10 days (p<0.05) while there were no statistically differences between other cell lines (**Figure 4**). This difference between the commercial AMSC line and primary AMSC line 654 suggests that inter-patient variability remains a concern with autologous stem cell lines; however, AMSC lines have similar proliferation capacity with BMSC lines indicates that AMSC may be used as therapeutic vehicles as well as BMSCs.

## Discussion

### AMSC and BMSC tumor tropism is similar *in vitro*

This is the first study to directly compare AMSC and BMSC tropism toward glioma-secreted factors in vitro. Furthermore, this is the first comparison of primarily obtained AMSCs directly from our patients which adds great novelty and potentially opens the doors for future personalized medicine for each patient. The results suggest that this tropism towards gliomas is similar between MSCs from bone marrow and adipose tissue sources in vitro. Given the previously documented similarities between MSCs from these two tissue sources, these results support the continued investigation of both cells as effective methods for selectively targeting gliomas.

### AMSC and BMSC cell proliferation rates

There were statistically significant differences between commercial BMSCs and commercial and primary culture AMSC lines. These differences were most notable between the commercial BMSC and primary culture AMSC lines, suggesting that primary cultures may not grow as robustly as their commercially obtained counterparts. Further research comparing primary culture BMSCs to primary culture AMSCs may be required prior to clinical usage of MSCs. Our observational data has indicated that while BMSCs and AMSCs proliferate at similar rates in the 24-well growth assay, BMSC demonstrate slower growth characteristics when grown in larger culture flasks and at lower seeding densities (not shown); this finding has been documented elsewhere in the literature [35]. The proliferative capacity of MSCs may be important not only in maximizing clinical applications, but also in minimizing the risk of oncogenic or malignant transformation, which has been linked to the length of *ex vivo* culture in some studies [33], [40]. This may influence the choice of an MSC source when transitioning to clinical applications, as large-scale expansion of individual patient MSCs will be necessary.

### Primary cell lines demonstrate inter-patient variability

There was no significant difference in tumor tropism between AMSCs and BMSCs. However, marked variability was noted between primary AMSC cell lines, indicating the potential for inter-patient variability in cells used for future clinical applications. Moreover, the glioma conditioned media from both primary glioma cell lines demonstrated variability in effecting MSC chemoattraction through secreted factors, emphasizing the need for future research into mechanisms underlying MSC tumor tropism.

### Adipose tissue offers a more efficient source of MSC, with reduced patient risk

Our data demonstrates that AMSCs and BMSCs have similar tumor tropism *in vitro*. However, while the volume of bone marrow that can be harvested under local anesthesia is calculated to provide 2.4×10^4^ MSC, the volume of adipose that can be harvested under local anesthesia is calculated to provide 1×10^6^ MSC [36]. This two fold difference in harvest volume makes adipose tissue the more efficient cell source (**Table 1**). Furthermore, the literature indicates that a higher seeding density is necessary for the successful growth and expansion of BMSC [41]. Although there was no statistically significant difference in proliferation rate between the AMSCs and BMSCs in our studies, other groups have found AMSCs to have a faster proliferation rate that is retained through multiple passages [42]. Without significant differences in tropism, AMSC should be considered as the preferred subset of MSC for continued clinical research.

**Table 1:** Description of harvesting and growth characteristics of adipose-derived and bone-marrow-derived mesenchymal stem cells.

	Adipose-Derived MSCs	Bone-Marrow-Derived MSCs
**Cells Harvested with Local Anesthesia ** ^[**44**]^	1×10^6^	2.4×10^4^
**Initial Seeding Density ** ^[**11**]^	1×10^4^	Required 10–20 fold greater than AMSC seeding density
**Population Doubling Time ** ^[**11]**, [35]^	78±26 h; 45.2 h	86±23 h; 61.2 h

### Limitations of experiments

These experiments provide data supporting similar tumor tropism between MSC sources, but there are limitations to the *in vitro* analysis. Although the matrigel migration assay and coated Boyden chamber invasion assay allow for the assessment of MSC migration and invasion through a synthetic extracellular matrix, it does not recapitulate the full range of substrates MSC must migrate through to travel from the peripheral circulation, traversing the blood brain barrier and brain parenchyma to home to glioma tumor bulk and microsatellite lesions. While the experiments in the present study suggest that AMSC and BMSC demonstrate similar glioma tropism *in vitro*, further studies using *in vivo* models are necessary to begin extrapolating these findings for clinical applications.

Given the inter-patient variability demonstrated in the primary AMSC lines, further investigation using additional primary AMSC lines and primary BMSC lines would provide information to guide the choice of the most efficient cell source for future clinical applications.

### Implication for future directions

A significant body of literature exists regarding the potential use of AMSC and BMSC as therapeutic delivery vehicles for patients with glioblastoma [43] [44] [45] [46] [47]. The ongoing research into potential therapeutic uses for MSC from a variety of tissue sources is predicated upon an assumption that AMSC and BMSC, given their similar cell surface marker expression profile and differentiation capacity, are equally efficacious at homing to glioma. While limited studies demonstrate similar homing efficacy between these two sources of MSCs in models of intracranial ischemia there have been no previous publications documenting similar glioma tropism *in vitro* or *in vivo*.

The present study is the first to demonstrate that *in vitro* tumor tropism is similar between AMSC and BMSC. These results support the ongoing research on both AMSC and BMSC as therapeutic delivery vehicles for intracranial glioma. However, as AMSCs may be harvested in greater numbers with less discomfort and morbidity, without differences in proliferation or glioma tropism, we recommend that adipose be considered the primary tissue source for MSCs in continued clinical research.

It must be noted that the mechanisms underlying this tropism may vary between different MSC sources. Further investigation is necessary to better define these mechanisms, so MSCs from different tissue sources may be effectively manipulated to deliver therapeutic molecules.

## Conclusions

Adipose- and bone marrow-derived mesenchymal stem cells appear to have similar tumor tropism *in vitro*. Given the well-documented ability to harvest larger numbers of AMSCs from adipose tissue obtained under local anesthesia, adipose tissue may prove to be a more efficient source of MSC for research and clinical applications, while minimizing patient morbidity during cell harvesting.
